# Supplementary material for: Comparative multiomics analysis of cell physiological state after culture in a basket bioreactor
Source: Sci Rep. 2022 Nov 23;12:20161. doi: 10.1038/s41598-022-24687-4 (PMC9686226; doi:10.1038/s41598-022-24687-4)
Supplement: Supplementary file 1 — Supplementary Information 1. [file 41598_2022_24687_MOESM1_ESM.zip › raw data/Proteomic raw data/9.PPI/EG.vs.CG/EG.vs.CG.StringDB_cut500.pdf]

EG.vs.CG

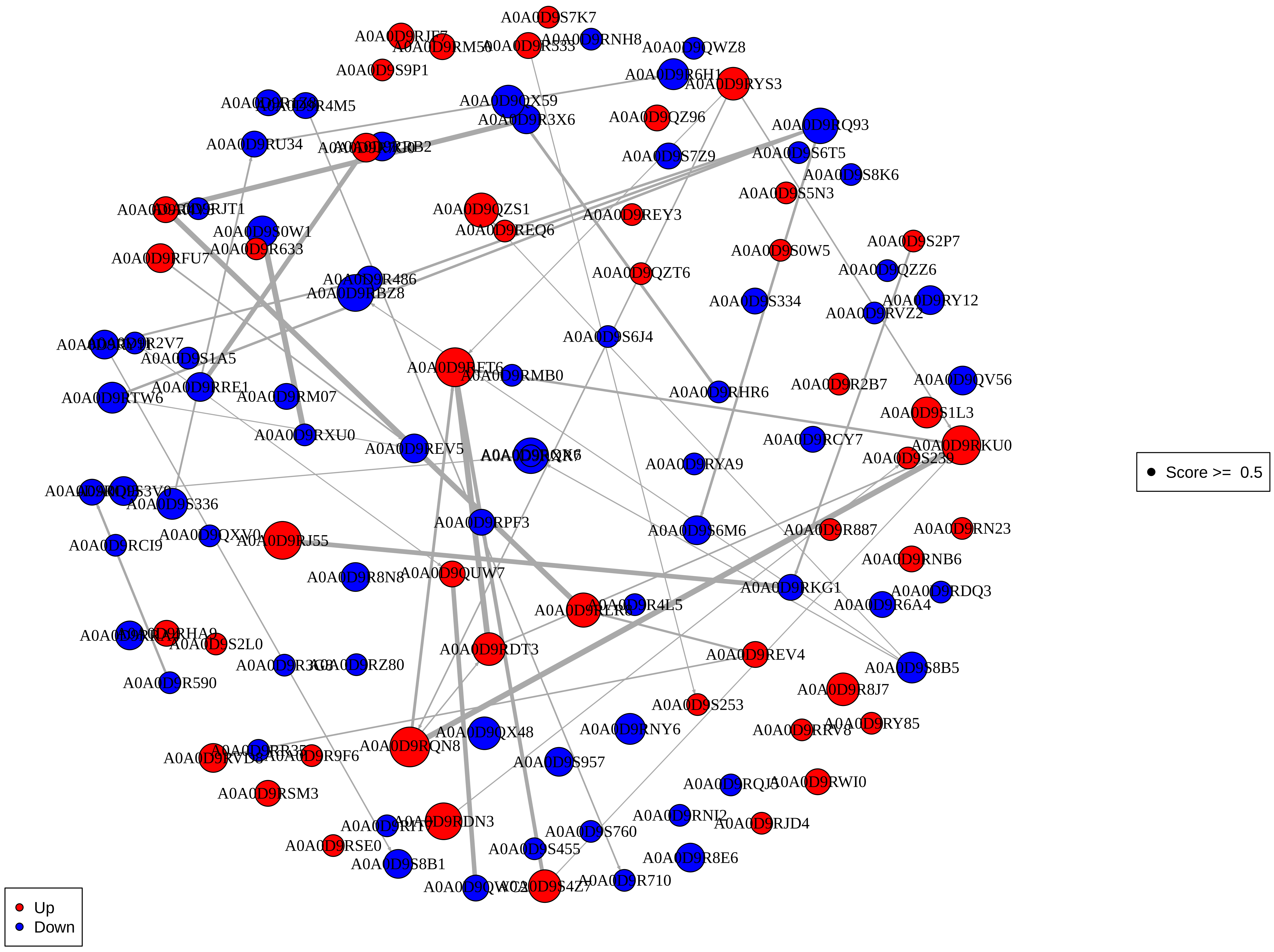
A0A0D9S7K7
A0A0D9RNH8
A0A0D9QWZ8
A0A0D9S9P1
A0A0D9R6H1
A0A0D9RYS3
A0A0D9QX59
A0A0D9R3X6
A0A0D9QZ96
A0A0D9RQ93
A0A0D9RU34
A0A0D9S7Z9
A0A0D9S6T5
A0A0D9S8K6
A0A0D9S5N3
A0A0D9QZS1
A0A0D9REQ6
A0A0D9REY3
A0A0D9RJT1
A0A0D9S0W1
A0A0D9R633
A0A0D9RFU7
A0A0D9S0W5
A0A0D9S2P7
A0A0D9QZZ6
A0A0D9QZT6
A0A0D9R486
A0A0D9RBZ8
A0A0D9S334
A0A0D9RY12
A0A0D9RVZ2
A0A0D9S6J4
A0A0D9S1A5
A0A0D9RMB0
A0A0D9RRE1
A0A0D9RTW6
A0A0D9RM07
A0A0D9RHR6
A0A0D9R2B7
A0A0D9QV56
A0A0D9S1L3
A0A0D9RXU0
A0A0D9REV5
A0A0D9RCY7
A0A0D9RKU0
A0A0D9S239
A0A0D9RYA9
A0A0D9S336
A0A0D9RPF3
A0A0D9S6M6
A0A0D9R887
A0A0D9RN23
A0A0D9RCI9
A0A0D9QXV0
A0A0D9RJ55
A0A0D9RNB6
A0A0D9R8N8
A0A0D9QUW7
A0A0D9RKG1
A0A0D9RDQ3
A0A0D9R6A4
A0A0D9R4L5
A0A0D9S2L0
A0A0D9RDT3
A0A0D9REV4
A0A0D9RZ80
A0A0D9R590
A0A0D9S8B5
A0A0D9R8J7
A0A0D9S253
A0A0D9RY85
A0A0D9RRV8
A0A0D9RNY6
A0A0D9QX48
A0A0D9RQN8
A0A0D9R9F6
A0A0D9S957
A0A0D9RSM3
A0A0D9RQJ5
A0A0D9RWI0
A0A0D9RDN3
A0A0D9RNI2
A0A0D9RJD4
A0A0D9S760
A0A0D9RSE0
A0A0D9S455
A0A0D9S8B1
A0A0D9R8E6
A0A0D9R710
A0A0D9S4Z7
Score >= 0.5
Up
Down
